# Modeling the MreB-CbtA Interaction to Facilitate the Prediction and Design of Candidate Antibacterial Peptides

**DOI:** 10.3389/fmolb.2021.814935

**Published:** 2022-01-27

**Authors:** Elvis Awuni

**Affiliations:** Department of Biochemistry, School of Biological Sciences, CANS, University of Cape Coast, Cape Coast, Ghana

**Keywords:** protein-protein interactions, PPI inhibitors, CbtA, MreB, modeling, antibacterial peptides, molecular dynamics simulations, antiMreB peptides

## Abstract

Protein-protein interactions (PPIs) have emerged as promising targets for PPI modulators as alternative drugs because they are essential for most biochemical processes in living organisms. In recent years, a spotlight has been put on the development of peptide-based PPI inhibitors as the next-generation therapeutics to combat antimicrobial resistance taking cognizance of protein-based PPI-modulators that interact with target proteins to inhibit function. Although protein-based PPI inhibitors are not effective therapeutic agents because of their high molecular weights, they could serve as sources for peptide-based pharmaceutics if the target-inhibitor complex is accessible and well characterized. The *Escherichia coli (E. coli)* toxin protein, CbtA, has been identified as a protein-based PPI modulator that binds to the bacterial actin homolog MreB leading to the perturbation of its polymerization dynamics; and consequently has been suggested to have antibacterial properties. Unfortunately, however, the three-dimensional structures of CbtA and the MreB-CbtA complex are currently not available to facilitate the optimization process of the pharmacological properties of CbtA. In this study, computer modeling strategies were used to predict key MreB-CbtA interactions to facilitate the design of antiMreB peptide candidates. A model of the *E. coli* CbtA was built using the trRosetta software and its stability was assessed through molecular dynamics (MD) simulations. The modeling and simulations data pointed to a model with reasonable quality and stability. Also, the HADDOCK software was used to predict a possible MreB-CbtA complex, which was characterized through MD simulations and compared with MreB-MreB dimmer. The results suggest that CbtA inhibits MreB through the competitive mechanism whereby CbtA competes with MreB monomers for the interprotofilament interface leading to interference with double protofilament formation. Additionally, by using the antiBP software to predict antibacterial peptides in CbtA, and the MreB-CbtA complex as the reference structure to determine important interactions and contacts, candidate antiMreB peptides were suggested. The peptide sequences could be useful in a rational antimicrobial peptide hybridization strategy to design novel antibiotics. All-inclusive, the data reveal the molecular basis of MreB inhibition by CbtA and can be incorporated in the design/development of the next-generation antibacterial peptides targeting MreB.

## Introduction

Protein-protein interactions (PPIs) are essential for most cellular and biochemical processes occurring in living organisms and thus have emerged as promising targets for the development of PPI modulators as alternative drugs (Loregian and Palù, 2005; Fry, 2015; Nevola and Giralt, 2015; Modell et al., 2016; Lee et al., 2019; Lu et al., 2020; Muttenthaler et al., 2021). Until recently, PPIs were considered as ‘undruggable’ targets because the interfaces involved in the interactions are mostly large, flat, and devoid of the conventional drug pockets (Jones and Thornton, 1996; Lo Conte et al., 1999; Hopkins and Groom, 2002). Following the advancement in strategies (Higueruelo et al., 2013) and the discovery of ‘hotspots’ as mostly responsible for the interaction and affinity between proteins (Clackson and Wells, 1995; Keskin et al., 2005; Cukuroglu et al., 2014), however, remarkable progress has been made in targeting PPIs and currently, there are approved PPI drugs with many other PPI inhibitors in clinical trials (Wells and Mcclendon, 2007; Nero et al., 2014; Scott et al., 2016; Lu et al., 2020). Therapeutic PPI modulators commonly come in the form of small molecules, antibodies, and peptides with each having its pros and cons. Although the peptide-based therapeutic PPI modulators are particularly disadvantaged by short half-lives and susceptibility to proteolytic degradation (Vlieghe et al., 2010), they are considered as prospective therapeutic agents because of their characteristic high target specificity and affinity, flexibility resulting in adaptability, multiple mechanisms, and nontoxic byproducts compared with the traditional organic molecules (Loffet, 2002; Recio et al., 2017).

There are many protein-based PPI modulators, made up of more amino acid residues than the 12–50 residues requirement for antimicrobial peptides, known to interact with their target proteins to inhibit function. On the basis of the assumption that only a few amino acid residues (hotspots) drive protein-protein complex formation, the pharmacological properties of such protein-based inhibitors could be optimized by applying strategies such as scaffold reduction and epitope transfer for size reduction to include only the important amino acid residues (Cunningham and Wells, 1997; Cochran, 2000). Such a rational protein-based PPI inhibitor size-reduction strategy could be expedited if a well-characterized complex of the pharmacological target and the inhibitor is available. The complex will thus reveal details about the interaction that could guide the size reduction or a peptidomimetic design process. Although it could be a daunting task to reduce a protein inhibitor to a short peptide and still conserve its binding and affinity characteristics, because the hotspots are usually not localized, and isolated short peptides may lose structural stability, some phenomenal successes have been made by applying these techniques (Braisted and Wells, 1996; Starovasnik et al., 1997; Domingues et al., 1999; Vita et al., 1999).

CbtA is a 124-amino-acid-residue toxin subunit of the CbtA/CbeA chromosomal toxin-antitoxin system of *Escherichia coli (E. coli)* and related species that is capable of inhibiting bacterial cell division and elongation by targeting FtsZ and MreB (Tan et al., 2011). It has been shown through mutation studies (Heller et al., 2017) that the *E. coli* CbtA interacts with the interprotofilament interface of the bacterial actin homolog MreB leading to the inhibition of cell elongation by possibly interfering with the polymerization of MreB monomers into double protofilaments (Van Den Ent et al., 2014) required for the determination and maintenance of cell shape and regulation of other important cellular processes in rod-shaped bacteria (Doi et al., 1988; Wachi and Matsuhashi, 1989; Jones et al., 2001; Kruse et al., 2003; Soufo and Graumann, 2003; Gitai et al., 2004; Gitai et al., 2005; Kruse and Gerdes, 2005; Soufo and Graumann, 2005). Hence, it has been suggested that CbtA has antibiotic properties and could be a useful source of antibacterial peptides targeting MreB (Heller et al., 2017; Awuni, 2020), which has been identified as a promising antibiotic target (Kruse et al., 2005; Vollmer, 2006; White and Gober, 2012; Awuni, 2020). Unfortunately, however, the three-dimensional (3D) structures of CbtA and the MreB-CbtA complex are not available to facilitate the optimization process of the pharmacological properties of CbtA. In this study, a possible MreB-CbtA complex was modeled to identify relevant interactions that could facilitate the development of antibacterial peptides against MreB. A 3D model of CbtA was built by using the trRosetta software (Yang et al., 2020) and its stability and dynamics were assessed through molecular dynamics (MD) simulations. The modeling and simulations data pointed to a model with reasonable quality and stability. By applying experimentally determined information regarding the MreB-CbtA interaction (Heller et al., 2017), the HADDOCK protein-protein docking software (Dominguez et al., 2003; De Vries et al., 2010) was used to predict a possible MreB-CbtA complex which was then characterized through MD simulations. The stability and other dynamics of the MreB-CbtA complex compared very well with MreB-MreB dimmer, suggesting that CbtA in its predicted binding mode could compete with MreB monomers for the interprotofilament interface of MreB. By using the antiBP software (Lata et al., 2007) to predict antibacterial peptides in CbtA, and the modeled MreB-CbtA complex as reference structure to determine important interactions and contacts, candidate antiMreB peptides were suggested. The data can be useful in the design/development of antibacterial peptides targeting MreB.

## Methods

### Retrieval and Preparation of Structures

The FASTA sequence of CbtA consisting of 124 amino acid residues was downloaded from the UniProt database (ID: P64524). On the other hand, the crystal structure of a double-protofilament *Caulobacter crescentus* MreB (CcMreB), bound to AMPPNP and Mg^2+^ (PDB ID: 4CZJ (Van Den Ent et al., 2014)), was downloaded from the RCSB Protein Data Bank. All water molecules were deleted and the SWISS-MODEL server (Guex et al., 2009; Waterhouse et al., 2018) was used to replace missing residues. AMPPNP, a nonhydrolyzable analog of ATP, was modified to ATP, which is the natural ligand. Then, the monomeric and dimeric forms of the ATP-Mg^2+^-bound CcMreB were constructed and used in this study. The dimmer involved monomers interacting laterally at the interprotofilament interfaces.

### CbtA Modeling and Model Selection

After using the CbtA sequence as the query in a BLAST (Altschul et al., 1990) search, no homologous structures were retrieved to support homology modeling of the 3D structure. Therefore, six popular ab initio-based modeling servers including CabsFold (Kolinski, 2004; Blaszczyk et al., 2013), Falcon (Wang et al., 2016), I-Tasser (Roy et al., 2010; Yang et al., 2015; Yang and Zhang, 2015), Quark (Xu and Zhang, 2012; Xu and Zhang, 2013), Robetta (Kim et al., 2004), and trRosetta (Yang et al., 2020); and three threading-based servers including Intfold (Mcguffin et al., 2019), Phyre2 (Kelley et al., 2015), and RaptorX (Peng and Xu, 2011) were employed to predict 3D models of CbtA. Nine models, constituted by the best model of each server, were selected and subjected to Ramachandran analyses (Ramachandran et al., 1963) using the PROCHECK program (Laskowski et al., 1993) to identify good structures. The outcome of the analyses showed that predicted models of Robetta, trRosetta, and RaptorX were of the best quality (Supplementary Figure S1 and Supplementary Table S1). Therefore, the server preference to predict the CbtA model was narrowed to Robetta, trRosetta, and RaptorX. For each of these three servers, twenty repeats of the modeling process were carried out and the results showed that while trRosetta and RaptorX were precise and returned the same 3D model in each run, Robetta generated different 3D folds of the protein. The 3D folds of the trRosetta and RaptorX models were similar; however, the trRosetta model had a higher percentage of amino acid residues in the favored regions of the Ramachandran plot than the RaptorX model (Supplementary Figures S1F, I and Supplementary Table S1). Thus, on the basis of the precision of model server and model quality, the trRosetta model was selected as the most reliable *E. coli* CbtA model for further studies.

### Molecular Dynamics Simulations of the CbtA Model

The conventional MD simulations protocol was applied by using the GROMACS 2020.4 simulation suite (Van Der Spoel et al., 2005). The topology of CbtA was built by using the Amber 99SB force field (Cornell et al., 1995) and the TIP3P water model (Jorgensen et al., 1983). The protein was placed at the center of a cubic box whereby 10 Å was specified as the minimum distance of separation between the surface of the protein and the edges of the box. After solvating the box with an explicit TIP3P water model (Jorgensen et al., 1983), the net charge of the system was neutralized and the ionic strength was set at 0.1 M by adding the required number of Na^+^ and Cl^−^ ions. Long-range electrostatic interactions were treated using the Particle Mesh Ewald method (Darden et al., 1993). The LINCS algorithm (Hess et al., 1997) was used to restrain all bonds involving hydrogen atoms at their equilibrium lengths, and the system was energy-minimized by using the steepest descent algorithm. By applying position restraints on the protein-heavy atoms, a 5 ns equilibration was carried out at 300 K using the NVT ensemble and the V-rescale thermostat (Bussi et al., 2007) after random initial velocities were assigned to the protein and solvent atoms. This was then followed by another 5 ns equilibration at 1 bar and 300 K in the NPT ensemble using the Berendsen barostat for pressure and the V-rescale thermostat (Bussi et al., 2007) for temperature coupling. The simulations were continued in the NPT ensemble to 150 ns with data collected every 1 ps. Three repeats of the simulations were carried out. The trajectory with the best cosine content was extended to 300 ns to further enhance conformational sampling.

### MreB-CbtA Docking

To predict a possible mode of interaction between MreB and CbtA, the monomeric ATP-Mg^2+^-bound CcMreB and a CbtA structure extracted from the deepest basin in a free energy landscape (FEL) analysis were used as the receptor and ligand models, respectively, for a protein-protein docking involving the HADDOCK 2.4 server (Dominguez et al., 2003; De Vries et al., 2010). The structures were reduced and then relaxed through energy minimization in GROMACS 2020.4 using the steepest descent algorithm. After uploading the two proteins to the HADDOCK program, the interacting interfaces were defined based on the findings of a previous study (Heller et al., 2017). Heller et al. (2017) showed that the interprotofilament interface of MreB is involved in the interaction with CbtA and demonstrated through mutation studies that amino acid residues I123, V170, E193, and E261 in CcMreB are important for the MreB-CbtA interaction. Residues F82, V118, and D189 were also considered important for the interaction. As a result, these amino acid residues of CcMreB were used as the active residues, and the surrounding residues on the flat face were used as the passive residues in the HADDOCK setup. In the same study (Heller et al., 2017), the amino acid residue R15 of CbtA was suggested to be important and required by CbtA to bind to MreB. Thus R15 was defined as an active residue and the surrounding residues were assigned as passive. The default HADDOCK settings were then used to dock the two proteins. After the docking process, the best binding mode out of the top five complex clusters was selected for analysis.

### MreB-CbtA and MreB-MreB Complex Simulations

The protocols for the simulations of the MreB-CbtA and MreB-MreB complexes were the same as CbtA. However, the topology of ATP was built for the general amber force field (GAFF) by using the antechamber utility in the AMBER 20 suite (Pearlman et al., 1995; Case et al., 2005) to assign atom types and RESP charges. Three repeats of 100 ns simulations were performed for each system.

## Results

### CbtA Modelling Results

**FIGURE 1:**
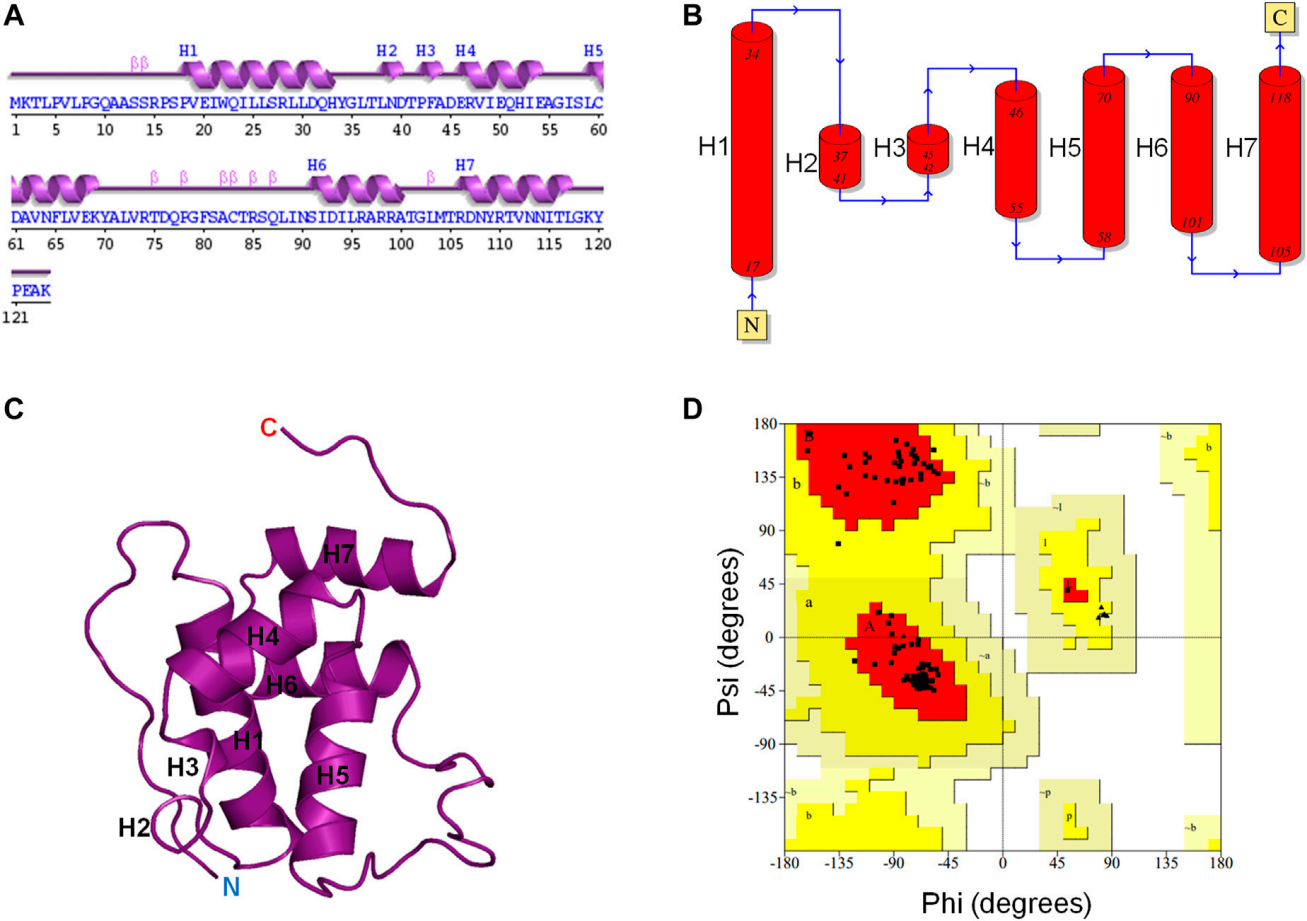
CbtA modeling data. **(A, B)** Assigned secondary structures to the amino acid residues of the CbtA model. The Arabic numerals represent the amino acid sequence numbers. H1, H4, H5, H6, and H7 are α-helices. H2 and H3 are 3_10_-helices. **(C)** The Predicted 3D fold of CbtA. **(D)** A Ramachandran plot showing the Phi (φ) and Psi (ψ) combinations of the amino acid residues in CbtA.

The 3D structure of the *E. coli* toxin CbtA is yet to be determined. Thus, to be able to establish a possible MreB-CbtA interaction to facilitate the process of predicting peptide-based inhibitors of the MreB-MreB interactions required to form the rod-shaped determining double protofilaments in bacteria (Van Den Ent et al., 2014), the 3D structure of CbtA was predicted using the trRosetta server (Yang et al., 2020). As illustrated in Figures 1A–C, the predicted CbtA model has seven helices consisting of five α-helices (H1, H4, H5, H6, and H7) and two short 3_10_-helices (H2 and H3). There are also β-turns, bends, and coils. The protein folds into a globular shape (Figure 1C), and the Ramachandran plot (Figure 1D) shows that 98.2% of the amino acid residues are found in the most favored regions colored red, and 1.8% in the additional allowed regions colored yellow. No non-glycine and non-proline amino acid residues were found in the generously allowed regions shown in wheat and the disallowed regions colored in white and indicate that the structure is of good quality. To assess the stability and dynamics of the CbtA model, MD simulations were carried out using GROMACS (Van Der Spoel et al., 2005).

### CbtA Model Simulation Results

#### System Validation and Convergence

**FIGURE 2:**
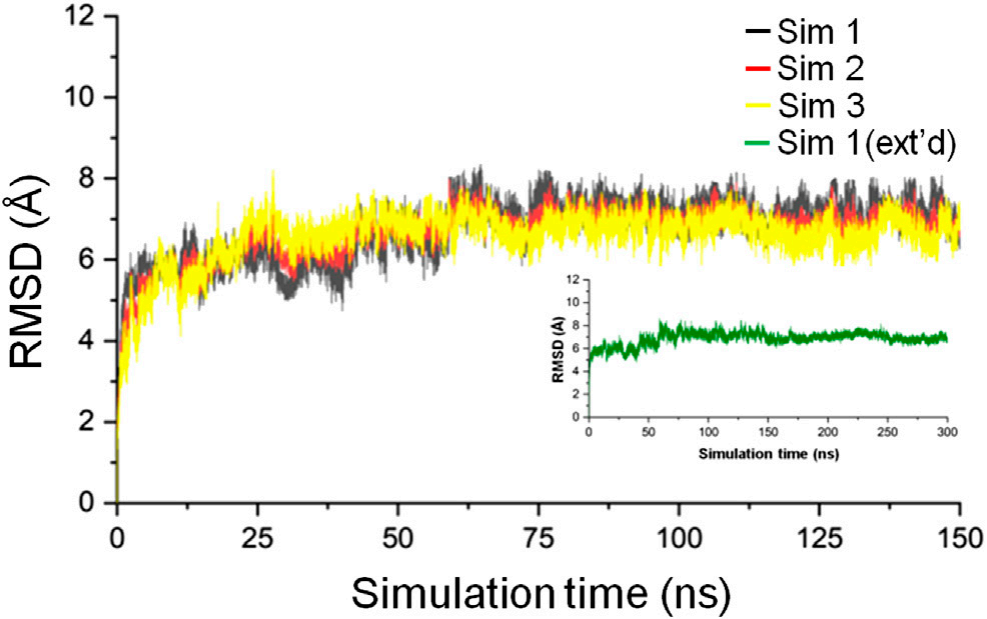
Backbone RMSD of the CbtA model. The black, red, and yellow curves represent the backbone RMSDs for simulations 1, 2, and 3, respectively. The green curve represents the backbone RMSD for the 300 ns extended (ext’d) simulation 1.

Three simulations, 150 ns each, of the CbtA model were carried out to check for the consistency and repetitiveness of the system. For each simulation, the initial structure was used as the reference and the gmx rms tool in GROMACS was used to calculate the root mean square deviation (RMSD) of the CbtA backbone as a function of time after least-square fitting to the backbone atoms. As illustrated in Figure 2, it can be observed that for the three simulations the backbone RMSDs of CbtA are close and convergence of each system is attained after 75 ns. The results suggest that at least the backbone conformational change of the model is consistent in the three systems. To find the trajectory with the most sampled CbtA conformations, the gmx analyze tool in GROMACS was used to calculate the cosine content from the first principal component generated by using the gmx covar and gmx anaeig utilities in GROMACS. The cosine content is reported as a value within the range of 0–1 where a lower value is indicative of a relatively better sampling for conformational analysis (Maisuradze and Leitner, 2007). From the calculations, cosine values of 0.544, 0.572, and 0.561 were obtained for simulations 1, 2, and 3, respectively. Thus, the trajectory of simulation 1 was extended to 300 ns to further explore the 3D space of the CbtA model. Figure 2 (inset) shows that the backbone RMSD of the model during the 300 ns simulations and indicates that the structure remains stable after 75 ns. Where appropriate, therefore, the last 225 ns of the 300 ns trajectory is used for analysis.

#### Model Stability Assessment

##### Hydrogen Bond Analysis

**FIGURE 3:**
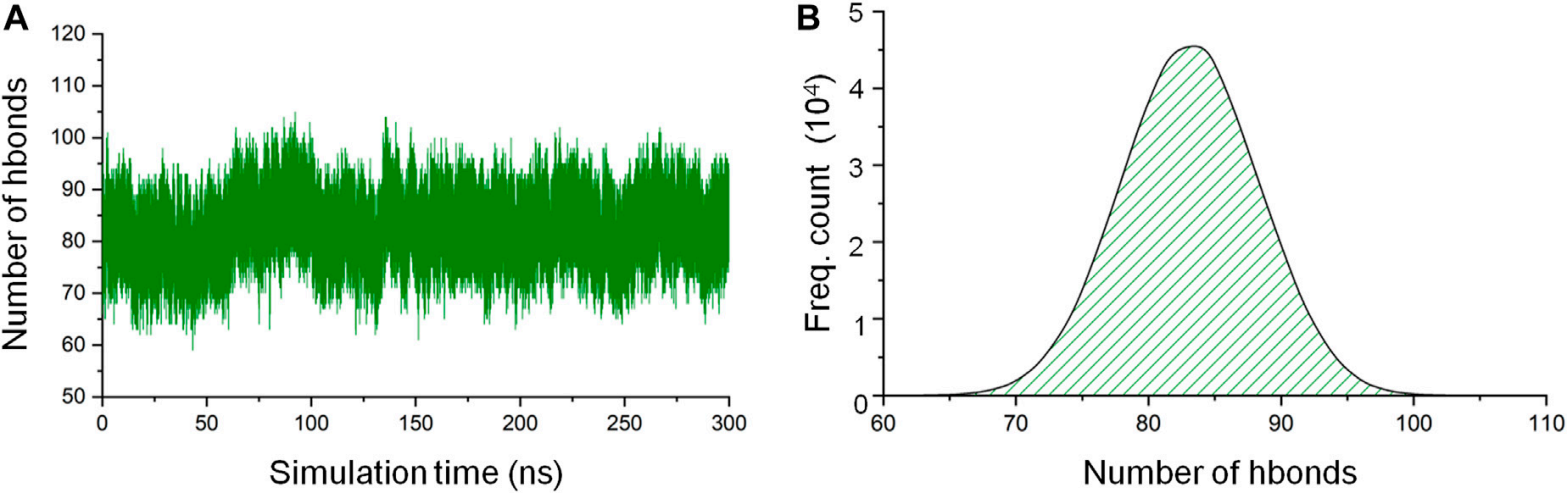
CbtA intramolecular hydrogen bond (hbond) analysis data. **(A)** Hydrogen bond count. **(B)** Hydrogen-bond-count distribution curve.

Hydrogen bonds are responsible for the formation of secondary structural elements and are also a major protein-3D structure stabilizing force. As one of the means to assess the stability of the CbtA model, the gmx hbond utility in GROMACS was used to calculate the total number of intramolecular hydrogen bonds within the protein as a function of time. The default values of a donor-acceptor distance of 0.35 nm and a donor-acceptor angle of 30° for defining a hydrogen bond were used in the calculations. It was observed that the hydrogen bond count remained fairly stable over the 300 ns simulations (Figure 3A), with the dominant and peak counts occurring within the ranges of 75–91 and 81–85, respectively, as illustrated by the hydrogen-bond-count distribution curve shown in Figure 3B. The results suggest that the model is stable, but it is important to find out if this stability relates to the secondary structures, 3D fold, or both.

##### Secondary Structure Analysis

**FIGURE 4:**
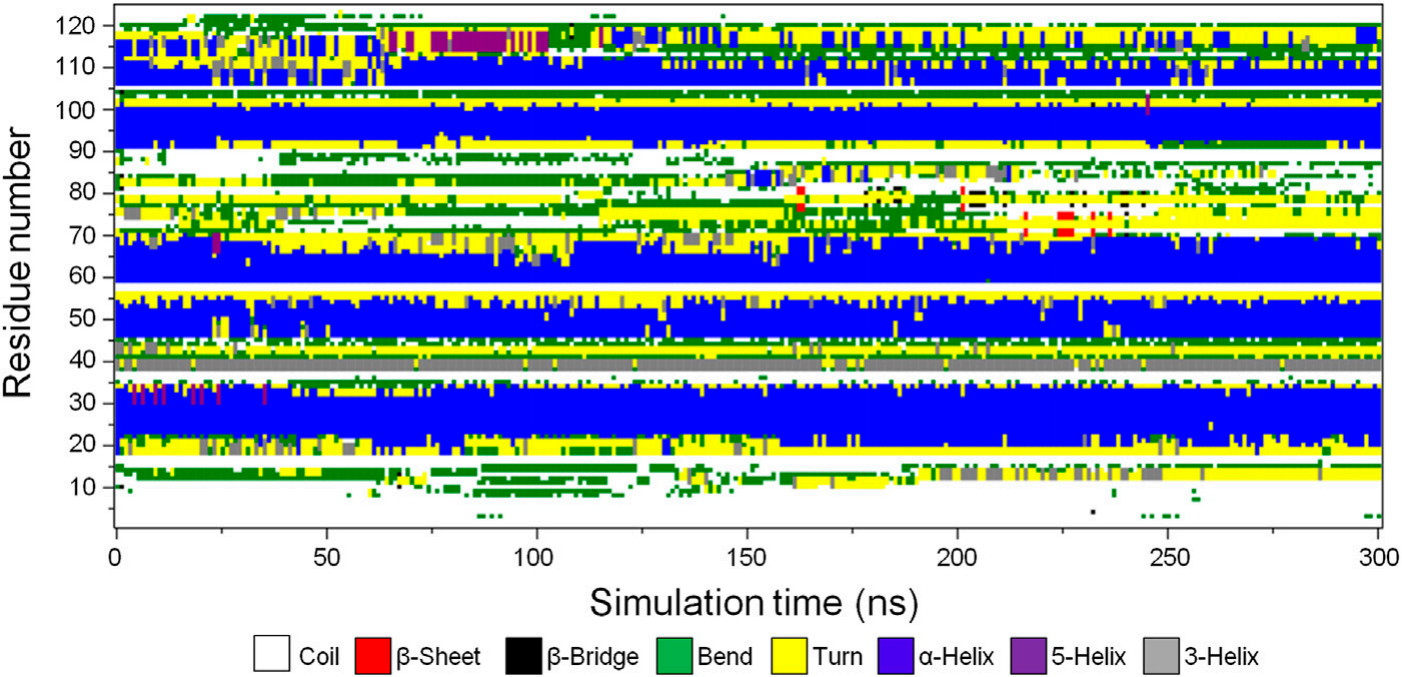
Secondary structure type assigned to the amino acid residues of CbtA. White, red, black, green, yellow, blue, purple, and gray colors represent coil, β-sheet, β-bridge, bend, turn, α-helix, 5-helix, and 3-helix, respectively.

To determine the stability of the secondary structure elements in the predicted CbtA model, the gmx do_dssp tool in GROMACS was used to monitor the evolution of secondary structures in the model over the 300 ns simulation time. Figure 4 and Supplementary Figure S2 illustrate the secondary structure type assigned to each of the 124 amino acid residues and the total number of residues involved in each secondary structure, respectively, as a function of time. Figure 4 and Supplementary Figure S2 show that the model is dominated by the following stable secondary structures in decreasing order of the number of amino acid residues involved: α-helices (blue), coils (white), turns (yellow), and bends (green); with some transient 3-helices (gray), 5-helices (purple), β-bridges (black), and β-sheets (red) occurring intermittently at some time steps in the course of the simulations. Overall, the results demonstrate that the major predicted secondary structure elements in the CbtA model are stable.

##### Radius of Gyration Analysis

**FIGURE 5:**
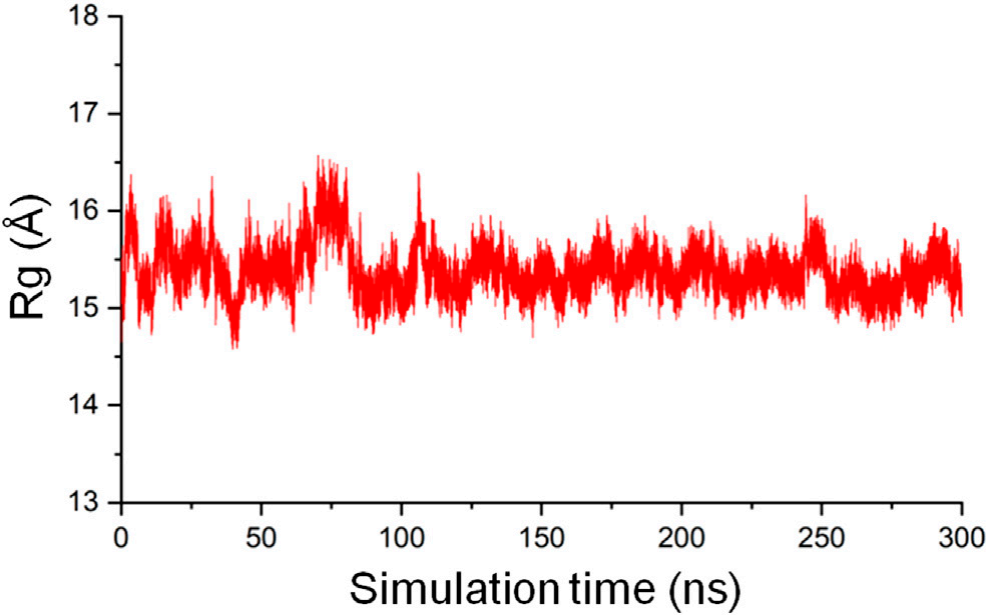
The radius of gyration of the CbtA-model backbone.

The radius of gyration (Rg) of a protein is the root mean square distance from each atom of the protein to their center of volume and is an indication of the compactness of the protein structure (Lobanov et al., 2008). In MD simulations, Rg as a function of time can be used to monitor the stability of the 3D fold of a protein on the basis that an unfolded structure will lead to an increased Rg, and a structure that gets tightly packed will result in a decreased Rg. The gmx gyrate tool in GROMACS was used to calculate the backbone Rg of the CbtA model as a function of time involving the 300 ns trajectory and the initial structure as the reference. The result, as illustrated in Figure 5, shows that the Rg is reasonably stable around 15.5 Å but sporadically fluctuates minimally between 14.5 Å and 16.5 Å, suggesting that the predicted 3D fold of the model remained stable during the 300 ns simulations.

#### Principal Component Analysis

**FIGURE 6:**
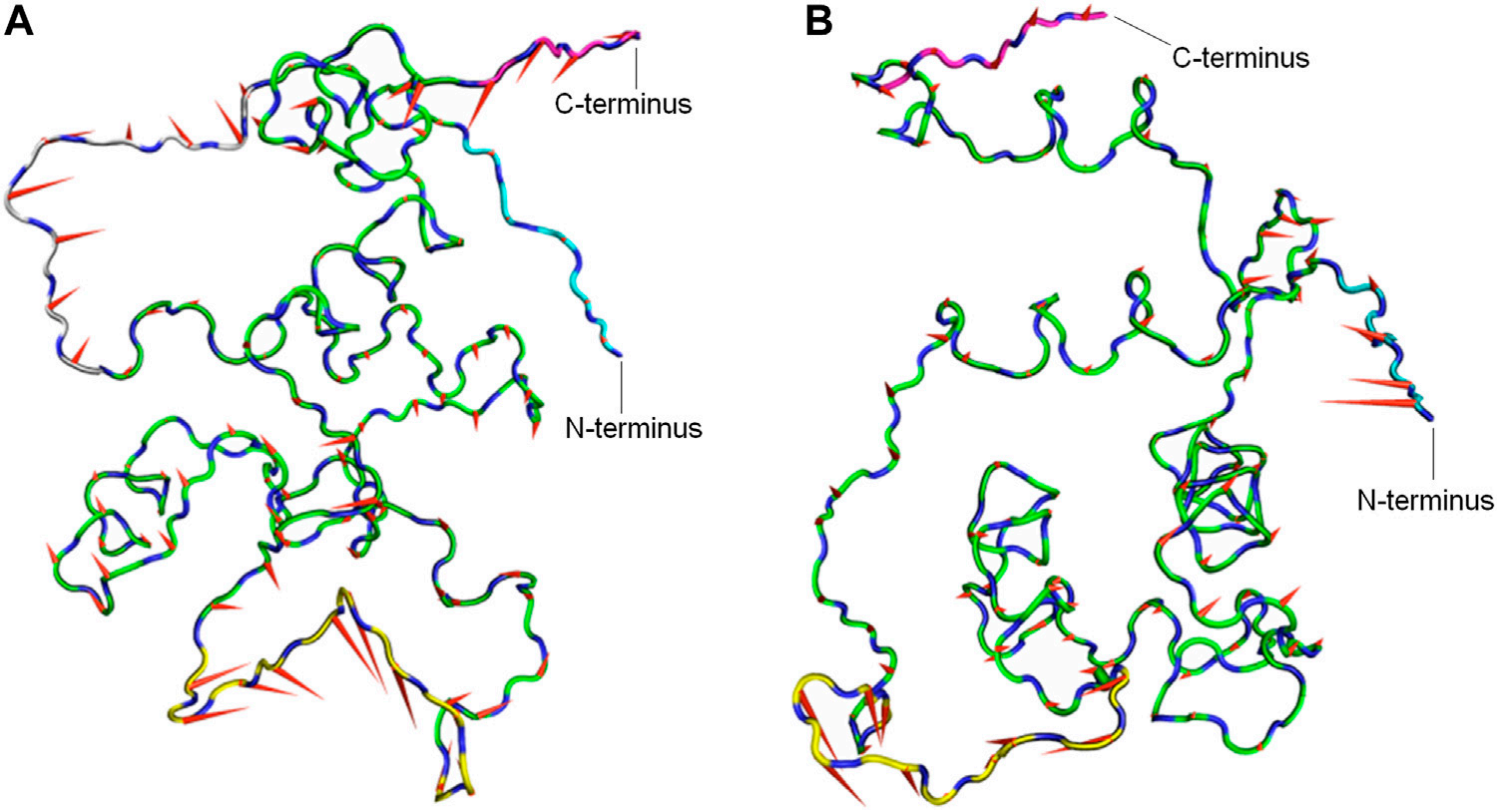
Porcupine plots from PCA analysis of the CbtA-model backbone. **(A)** Porcupine plot for PC1. **(B)** Porcupine plot for PC2. Regions colored yellow, gray, cyan, and magenta represent the T75-T84 segment, Q10-P16 segment, N-terminus, and C-terminus, respectively. The tip and length of each cone indicate the direction of the eigenvector and the magnitude of the corresponding value, respectively.

To determine the dominant motions in the backbone atoms of the CbtA model, principal component analysis (PCA) was performed on the backbone atoms using the gmx covar and gmx anaeig tools in GROMACS and the last stable 225 ns trajectory. The gmx covar tool was used to generate eigenvectors that showed that the first two eigenvectors accounted for all the significant motions in the model. Thus the gmx anaeig tool was then used to extract the extreme motions from the first two eigenvectors for the generation of porcupine plots to visualize the backbone motions. Figures 6A,B show the porcupine plots generated for the first and second principal components (PC1 and PC2), respectively. In the porcupine plots, the tip and length of each cone indicate the direction of the eigenvector and the magnitude of the corresponding value, respectively. The porcupine plot for PC1 (Figure 6A) indicates that the main motions in the CbtA backbone occur in the regions encompassing residues T75-T84 (yellow), Q10-P16 (gray), and the C-terminus (magenta). The plot for PC2 (Figure 6B) also reveals relevant motions in the N-terminus region (cyan). Interestingly these observed backbone motions are consistent with the backbone root mean square fluctuation (RMSF) data presented in Supplementary Figure S3 where two main peaks, corresponding to the amino acid residues Q10-P16 and T75-T84, and some fluctuations at the N- and C-termini were noticed. These regions represent the most flexible regions of the model; and it is not surprising because the Q10-P16 and T75-T84 segments are part of the N-terminal loop and the H5-H6 loop, respectively (Figure 1).

#### Free Energy Landscape Analysis

**FIGURE 7:**
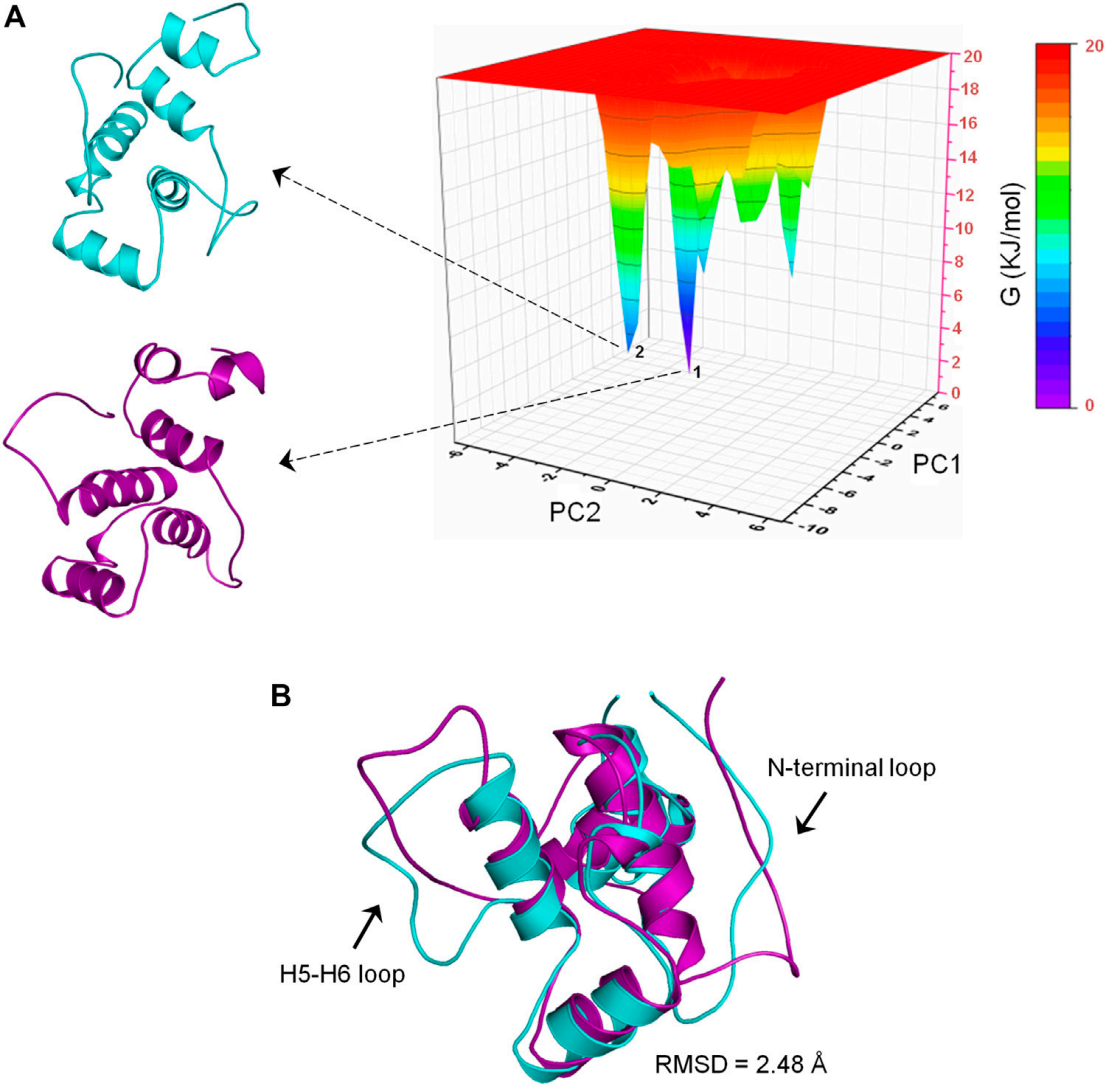
Free energy landscape (FEL) analysis for the CbtA model. **(A)** FEL diagram for CbtA showing structures extracted from the main minima. The structures are extracted from the tips of the wells which represent the most stable conformations. **(B)** Superimposed structures from minima 1 and 2 revealing the main structural variations. Structures from minima 1 and 2 are colored purple and cyan, respectively.

To determine the most energetically stable conformation(s) explored by CbtA during the simulations, FEL analysis was performed. The gmx anaeig tool was used to generate a projection of PC1 and PC2 which was then used to generate a 3D FEL plot as shown in Figure 7A. As indicated in Figure 7A, two main minima (basins 1and 2) were observed whereby basin 1 is the deepest and most populated and indicative of the most stable conformation. Superimposition of the backbone atoms of the structures extracted from the two minima gives RMSD of 2.48 Å with the main difference based on the construction of the N-terminal and H5-H6 loops (Figure 7B). The N-terminal and H5-H6 loops were identified in the PCA (Figure 6) and RMSF (Supplementary Figure S3) analyses as mobile and flexible regions of the protein. Accordingly, the structure extracted from basin 1 was selected for further studies. Structure quality assessment including verify 3D (Bowie et al., 1991; Lüthy et al., 1992) and ERRAT (Colovos and Yeates, 1993) analyses carried out on the extracted conformation pointed to a good structure with 83.87% of the residues having averaged 3D-1D score≥0.2 and an ERRAT overall quality score of 92.79%, respectively.

##### MreB-CbtA Docking Results

**FIGURE 8:**
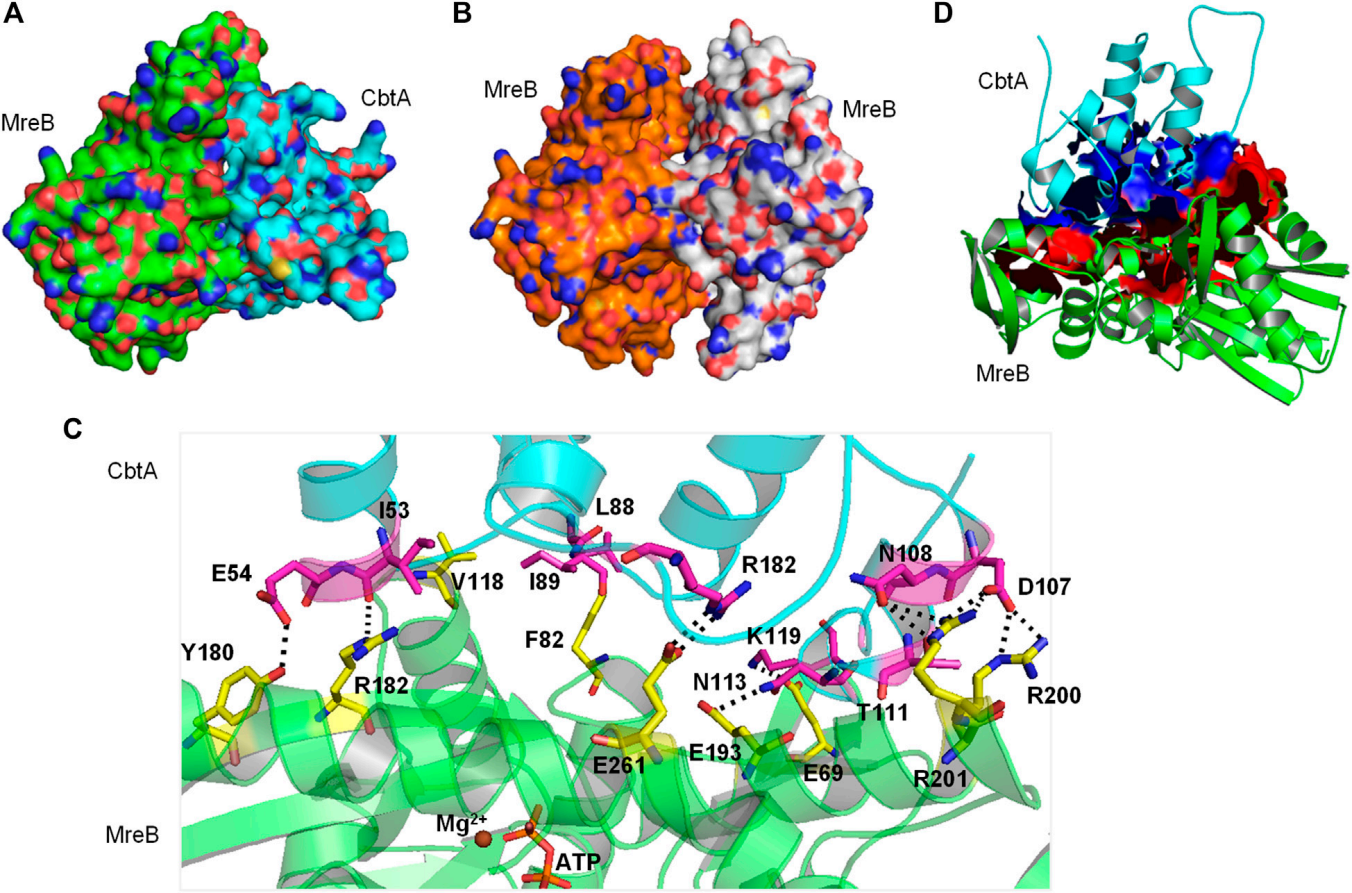
The MreB-CbtA interaction data. **(A)** Space-filling model of the MreB-CbtA complex. Carbon atoms of MreB and CbtA are colored green and cyan, respectively. **(B)** Space-filling model of the MreB-MreB complex. Carbon atoms of Chain A and Chain B are colored orange and gray, respectively. **(C)** Key interactions at the MreB-CbtA interface. Carbon atoms of MreB and CbtA are colored green and cyan, and the carbon atoms of the corresponding amino acid residues involved in the interactions are colored yellow and magenta, respectively. Oxygen and nitrogen atoms are colored red and blue, respectively. Hydrogen bonds are illustrated by black dashes. **(D)** Phobic interactions at the MreB-CbtA interface. Carbon atoms of MreB and CbtA are colored green and cyan, respectively. Atoms constituting the phobic interfaces of MreB and CbtA are colored red and blue, respectively.

A space-filling representation (Figure 8A) of the predicted MreB-CbtA complex shows that the interface presented by CbtA for the interaction fits the interprotofilament interface of MreB and occupies a significant surface area. The space-filling model of the CcMreB dimmer is shown in Figure 8B for comparison. The predicted MreB-CbtA complex is characterized by several interactions at the contacting interface (Figure 8C). Interestingly, most of the amino acid residues mentioned by Heller et al. (2017) as important for the MreB-CbtA interaction including F82, V118, V170, D189, E193, and E261 from MreB and R15 from CbtA are involved in important interactions in the predicted complex. The amino acid residues F82 and V118 from MreB are involved in hydrophobic interactions with L88 and I89 from CbtA; E193 from MreB forms a hydrogen bond with N113 from CbtA, and E261 from MreB forms a salt bridge with R15 from CbtA. Other important interactions observed include hydrogen bonds between E69 from MreB and K119 from CbtA; Y180 from MreB and E54 from CbtA; R182 from MreB and I53 of CbtA; and a network of hydrogen bonds between R200 and R201 from MreB and D107, N108 and T111 from CbtA. Also, the contacting interfaces presented by both MreB and CbtA possess the hydrophobic patches, shown as red and blue surfaces respectively in Figure 8D, required to drive PPIs. PPIs are mainly driven by the hydrophobic effect resulting from the dislodging of water molecules bound to hydrophobic patches at the contacting interfaces leading to an increase in entropy and the generation of favorable Gibbs free energy (Tsai et al., 1997; Southall et al., 2002; Chanphai et al., 2015).

##### MreB-CbtA Complex Stability Assessment

**FIGURE 9:**
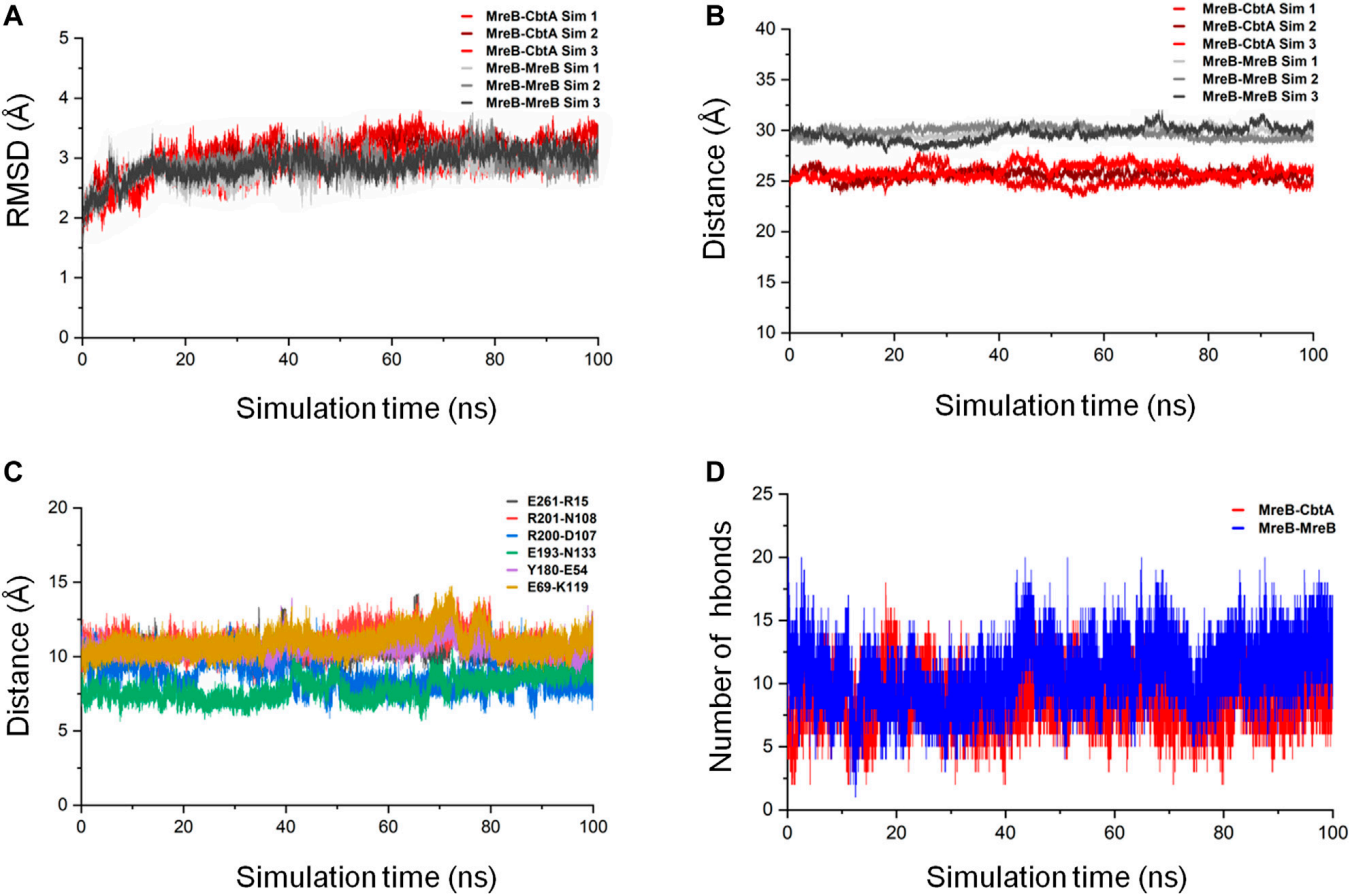
MreB-CbtA and MreB-MreB complex simulation analyses. **(A)** Backbone RMSDs of the MreB-CbtA and MreB-MreB complexes. The backbone RMSDs for the three MreB-CbtA and three MreB-MreB complex simulations are illustrated by the colors in red shade and gray shade, respectively. **(B)** Distances between the center of masses (COMs) of MreB and CbtA, and the MreB pair. The distances between the COMs of the MreB and CbtA, and MreB and MreB complexes for the three simulations in each case are illustrated by the colors in red shade and gray shade, respectively. **(C)** The distances between the COMs of the α-Carbon (CA) atoms of the amino acid residue pairs (E261-R15, R201-N108, R200-D107, E193-N133, Y180-E54, and E69-K119) involved in hydrogen bonding between MreB and CbtA. **(D)** The intermolecular hydrogen bonds (hbonds) between the interacting pairs (MreB-CbtA, and MreB-MreB). The red curve shows the number of hydrogen bonds between MreB and CbtA. The blue curve shows the number of hydrogen bonds between the two MreB subunits.

To ascertain the stability of the MreB-CbtA complex, three repeats of 100 ns MD simulations were carried out. Similar simulations were conducted involving the MreB-MreB dimmer for comparison. The backbone RMSD of each complex was calculated as the first step to monitor stability and conformational change. By using the initial structures as references, the gmx rms tool in GROMACS was used to calculate the RMSD on the backbone atoms as a function of time after least-square fitting to the same atoms. The results of the backbone RMSD calculations in all six simulations (three each for MreB-CbtA and MreB-MreB complexes) are shown in Figure 9A where the RMSDs obtained from the three MreB-CbtA and three MreB-MreB complex simulations are illustrated by the colors in red and gray shades, respectively. Overall, the results for both MreB-CbtA and MreB-MreB complexes show backbone RMSDs not exceeding 3.5 Å that remain reasonably stable within this range during the simulations. Where necessary, the trajectory with the most stable backbone RMSD in each case was selected for analysis.

The proximity of proteins to each other is important for interaction (Yen et al., 2014; Peng et al., 2016); and where the interaction is weak or energetically unstable, the interacting subunits could separate during simulations leading to an increase in the separating distance. On this premise, the distances between the center of masses (COMs) of MreB and CbtA, and the MreB pair were measured as a function of simulation time by using the gmx distance tool in GROMACS. The results as illustrated in Figure 9B show that the distances between the COMs of the interacting partners are reasonably stable over the 100 ns simulations. The difference between the observed distances between the COMs of the MreB-CbtA (red color shades) and MreB-MreB (gray color shades) pairs is attributable to the fact that CbtA is relatively smaller (124 amino acids) than MreB (334 amino acids) and thus the distance between the COMs of MreB and CbtA is shorter than the distance between the COMs of the MreB-MreB pair. Also, the distances between the COMs of the α-Carbon (CA) atoms of the amino acid residue pairs involved in hydrogen bonding between MreB and CbtA including E261-R15, R201-N108, R200-D107, E193-N133, Y180-E54, and E69-K119 (Figure 8C) were measured as a function of simulation time. The results are presented in Figure 9C and suggest that the distances between the COMs of the CA atoms of the aforementioned residue pairs are reasonably stable during the100 ns simulations. Furthermore, to confirm that at least the key interactions between MreB and CbtA are stable, the intermolecular hydrogen bonds were calculated as a function of time by using the gmx hbond utility in GROMACS and compared with the MreB-MreB complex. Figure 9D shows that the hydrogen bond interactions between MreB and CbtA are fairly stable and compare well with the MreB-MreB complex.

**FIGURE 10:**
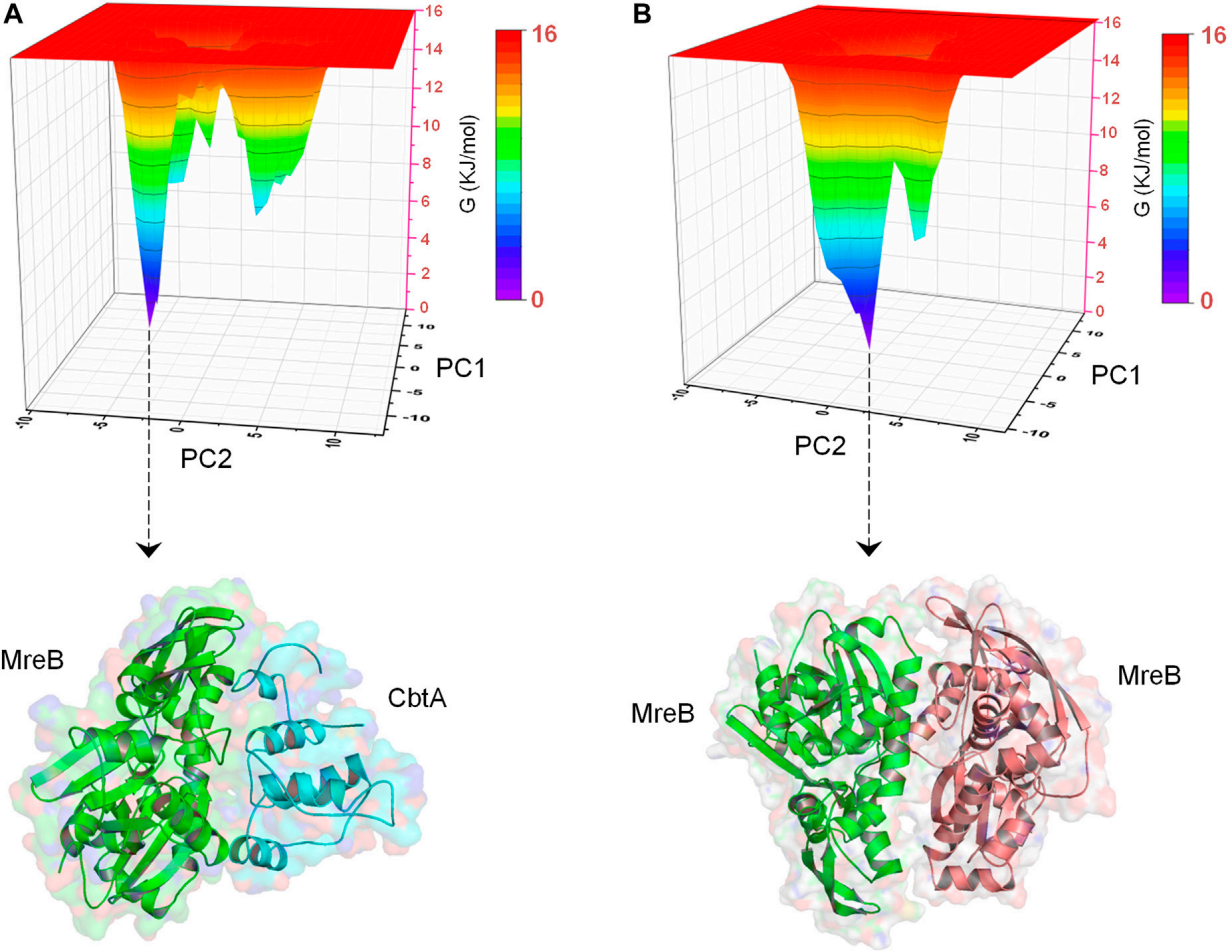
Free energy landscape (FEL) analyses for the MreB-CbtA and MreB-MreB complexes. **(A)** FEL diagram for the MreB-CbtA complex. **(B)** FEL diagram for the MreB-MreB complex. The structures are extracted from the tips of the wells which represent the most stable conformations.

In addition, to obtain energetically stable structures explored during the simulations to further confirm that the MreB-CbtA and MreB-MreB complexes are still intact, FEL analyses were performed on the backbone atoms by using the last 50 ns trajectories. Figures 10A,B show the FEL diagrams generated for the MreB-CbtA and MreB-MreB complexes and the corresponding structures extracted from the energy minima, respectively. As illustrated in Figures 10A,B, one main energy minimum was observed for each complex; suggesting that both complexes are stable as one energetically most-favorable conformation is prominent in each case. The representative structures extracted from the energy minima (Figures 10A,B) also demonstrate that both complexes remained intact during the 100 ns simulations. Unfortunately, in the current setup, it was difficult to make any valuable inferences by comparing the binding energies and the energetic contributions of the amino acid residues of the two systems as the results obtained in each case varied greatly over dielectric constants that span within and several folds above the recommended 1–40 range for most proteins (Fogolari et al., 2003; Li et al., 2013; Du et al., 2018; Sheng et al., 2021; Williams-Noonan et al., 2021). Nonetheless, the overall complex simulation data indicate that the MreB-CbtA complex remained stable over the 100 ns simulations, and suggest that CbtA could compete with MreB monomers for binding to the interprotofilament interface of MreB.

##### Prediction of Candidate AntiMreB Peptide Sequences

The CbtA length of 124 amino acid residues exceeds the required length of 12–50 residues for antimicrobial peptides. As suggested earlier (Awuni, 2020), it is possible to apply strategies to determine the minimum effective peptide lengths in CbtA responsible for the inhibition of MreB. To do this, herein the antiBP server (Lata et al., 2007) was used to predict the antibacterial peptides in the CbtA sequence. By using the QM, ANN, and SVM methods as well as the N-terminus, C-terminus, and NC-terminus strategies incorporated into the server, many peptide fragments were generated and the antibacterial possibility of each was indicated with a YES/NO flag. By using the MreB-CbtA-complex model as the reference, the predicted peptides that made contacts with the interprotofilament interface of MreB were selected. Table 1 shows eight peptide sequences that were selected as antiMreB peptide candidates or precursors for the design of antiMreB peptides. The contacting sites of the eight peptides on MreB are illustrated in Supplementary Figure S4.

**TABLE 1:** Predicted antiMreB peptide sequences.

Peptide	Sequence	Strategy (terminus)	Method	Hydrogen bonding partners in MreB
1	TINNVTRYNDRTMLG	C	SVM	Glu193, Arg200, Arg201
2	PVLPGQAASSRPSPV	N	ANN	Glu261
3	NNVTRYNDRTMLGTA	C	ANN	Glu193, Arg200, Arg201
4	EPYKGLTINNVTRYN	C	ANN	Glu193, Arg201
5	RLIDISNILQSRTCA	C	ANN	-
6	TLPVLPGQAASSRPS	N	QMM	Glu261
7	YKGLTINNVTRYNDR	C	QM	Lys119,Glu193, Arg200, Arg201
8	RSSAAQGPLVPLTKM	C	QM	Glu261

## Discussion and Future Prospects

Antimicrobial peptides are considered the best alternative as the new generation of therapeutic agents following their broad-spectrum and intrinsic ability to overcome multidrug-resistant strains (Mwangi et al., 2019; Moretta et al., 2021). The traditional *de novo* design and screening techniques in drug discovery are still useful in finding antimicrobial peptides; however, these techniques are confronted with challenges regarding the discovery of PPI inhibitors following the physicochemical properties of the targeted interfaces (Jones and Thornton, 1996; Lo Conte et al., 1999; Hopkins and Groom, 2002). This study predicts the 3D structures of CbtA and the MreB-CbtA complex and relies on the idea that a protein-receptor and protein-inhibitor complex is predominantly stabilized by a few key interactions to predict possible peptide inhibitors by a computational-based scaffold reduction strategy. This idea has been used in other techniques to effectively reduce the sizes of protein-based inhibitors of PPIs (Braisted and Wells, 1996; Starovasnik et al., 1997).

The predicted MreB-CbtA complex reveals interactions involving important amino acid residues from both partners. These interactions suggest a mechanism by which CbtA inhibits MreB as follows: The interaction between MreB and CbtA is driven by a phobic effect generated by the hydrophobic patches presented by the two interacting interfaces (Figure 8D). CbtA then makes strong interactions, including a network of hydrogen bonds and salt bridges, with important residues on the interprotofilament interface of MreB (Figure 8C). These features give the MreB-CbtA complex stability that is comparable with the MreB-MreB complex as demonstrated by the results of the RMSD (Figure 9A), distances (Figures 9B,C), intermolecular hydrogen bonds (Figure 9D), and FEL (Figures 10A,B) analyses obtained herein. In consequence, CbtA can compete with MreB monomers for the interprotofilament interface resulting in competitive inhibition of MreB-MreB dimerization.

**FIGURE 11:**
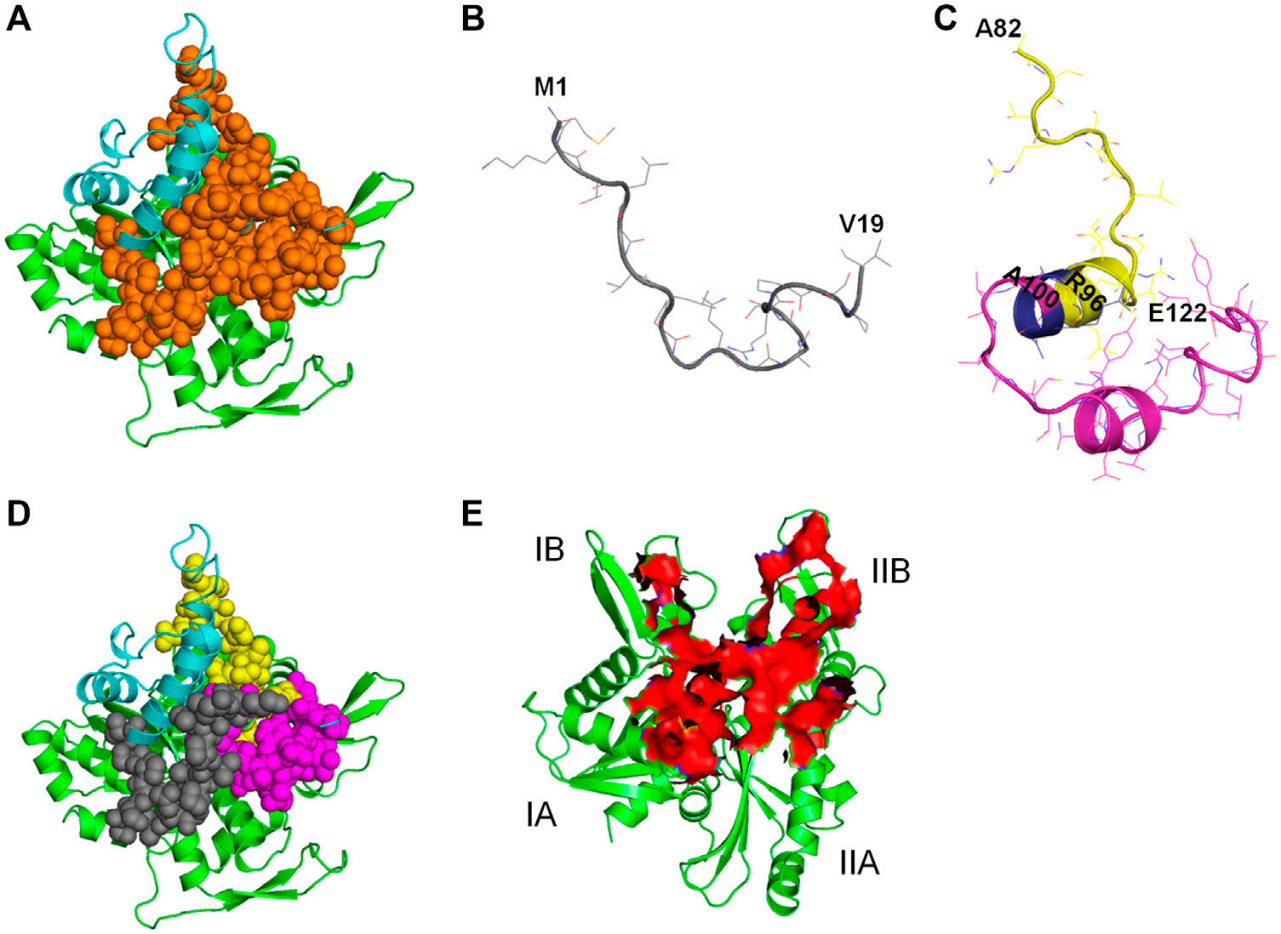
Illustration of antiMreB peptides design possibilities. Carbon atoms of MreB are shown in green. Carbon atoms of CbtA are colored in cyan. **(A)** Illustration of the polypeptide unit formed by the 8 predicted peptides on the interprotofilament interface of MreB. Orange spheres represent the continuous polypeptide unit formed by the eight predicted peptides in CbtA. **(B)** The M1-V19 segment. **(C)** The A82-R96 and A100-E122 segments shown in yellow and magenta, respectively. Blue shows the A97-R98-R99 tripeptide separating the two segments. **(D)** Illustration of the M1-V19 (gray), A82-R96 (yellow), and A100-E122 (magenta) segments on the interprotofilament interface of MreB. **(E)** Predicted region of MreB that harbors the hotspots for antibacterial peptides discovery/design.

All the 8 selected antibacterial peptide candidates have 15 amino acid residues each (Table 1), and well within the 12–50 residues requirement for an antimicrobial peptide; and cluster into a well-defined continuous polypeptide unit in the CbtA 3D structure as illustrated by the orange spheres in Figure 11A. The polypeptide unit is constituted by the M1-V19 (Figure 11B), A82-R96, and A100-E122 (Figure 11C) segments colored gray, yellow, and magenta, respectively, as shown in Figure 11D. It is thus suggested that the antiMreB property of CbtA resides in this polypeptide unit and that it is possible to transform this unit into a more potent inhibitor through a rational peptide hybridization strategy. Antimicrobial peptide hybridization which usually involves combining key residues from two or more peptides to form a novel therapeutic peptide sequence or improve certain properties has been reported (Fox et al., 2012; Almaaytah et al., 2018; Wang et al., 2019). Also, the A82-R96 and A100-E122 segments are linked by a short A97-R98-R99 tripeptide, colored blue in Figure 11C, and could be transformed into a continuous polypeptide chain with antibacterial activity. Further, three of the peptides including peptides 1, 3, and 7 predicted by the antiBP make useful hydrogen bonds and salt bridges with E193, R200, and R201 on the MreB interprotofilament interface (Table 1) and require further investigations to ascertain their inhibitory and pharmacokinetic properties. Interestingly all these three peptides contain the ‘NNVTRYNDR’ amino acid string and represent good candidates for optimization through rational peptide hybridization.

Hotspot amino acids play critical roles in PPIs and their mutations lead to the disruption of the interactions. In drug discovery involving PPIs, these hotspots help to narrow down the section of the interacting interface that could be targeted for the discovery and design of inhibitors (Keskin et al., 2005; Cukuroglu et al., 2014). It was observed herein that the residues of the interprotofilament interface of MreB that make important contacts with CbtA are located within the region shown as a red surface in Figure 11E. The residues within this region span the domains IA (including some residues from the dimerization helix which play a critical role in the double protofilament formation of MreB), IB, and IIB, with few from IIA. It is further suggested that this section of MreB harbors the hotspot amino acids and could play a critical role in a focused design of antiMreB peptides.

## Conclusion

Protein-based PPI inhibitors are common but have not been useful therapeutic agents because of their high molecular weight. Nonetheless, these inhibitors could serve as handy sources of information for the development of antimicrobial peptides if the target protein has pharmacological relevance and the target-inhibitor complex is available and well characterized. It was sought to use in silico techniques to predict and characterize the 3D structure of CbtA and a possible MreB-CbtA complex, and use the information obtained thereafter to suggest potential antiMreB peptide sequences. The results hint that CbtA inhibits MreB through the competitive mechanism driven by the key interactions CbtA makes with the interprotofilament interface of MreB resulting in the disruption of its polymerization into double protofilaments. Candidate antiMreB peptides that could also be integrated into a rational antimicrobial peptide hybridization strategy to design novel antibiotics were proposed.

## Data Availability

The datasets presented in this study can be found in online repositories. The names of the repository/repositories and accession number(s) can be found in the article/Supplementary Material.
