# Supplementary material for: Modeling the MreB-CbtA Interaction to Facilitate the Prediction and Design of Candidate Antibacterial Peptides
Source: Front Mol Biosci. 2022 Jan 27;8:814935. doi: 10.3389/fmolb.2021.814935 (PMC8828653; doi:10.3389/fmolb.2021.814935)
Supplement: Supplementary file 2 [file DataSheet1.DOCX]

Supplementary Material

# Supplementary Tables and Figures


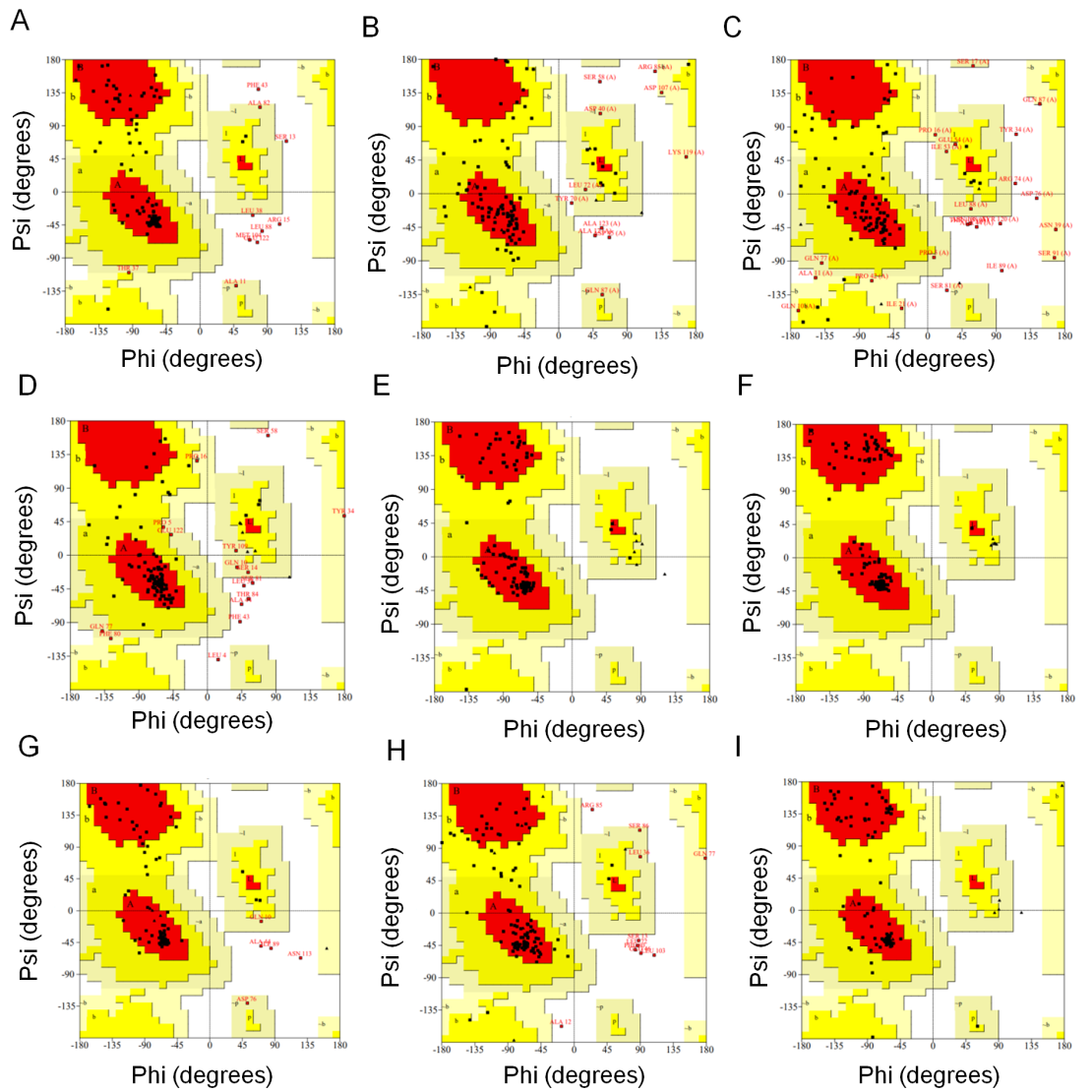


**Supplementary Figure S1.** Ramachandran analyses data. **(A)** Ramachandran plot for the CabsFold model. **(B)** Ramachandran plot for the Falcon model. **(C)** Ramachandran plot the for I-Tasser model. **(D)** Ramachandran plot for the Quark model. **(E)** Ramachandran plot for the Robetta model. **(F)** Ramachandran plot for the trRosetta model. **(G)** Ramachandran plot for the Intfold model. **(H)** Ramachandran plot for the Phyre2 model. **(I)** Ramachandran plot for the RaptorX model. The red, yellow, wheat, and white zones represent the most favored, additional allowed, generously allowed, and disallowed regions, respectively. The % of amino acid residues in each region is presented in Supplementary Table S1.

.

**Supplementary Table S1**: Distribution of amino acid residues in the Ramachandran plots of the nine CbtA models.

| Model | % Residues in most favored regions | % Residues in additional allowed regions | % Residues in generously allowed regions | % Residues in disallowed regions |
| --- | --- | --- | --- | --- |
| CabsFold | 78.9 | 11.9 | 2.8 | 6.4 |
| Falcon | 55.0 | 34.9 | 4.6 | 5.5 |
| I-Tasser | 55.0 | 26.6 | 10.1 | 8.3 |
| Quark | 68.8 | 18.3 | 6.4 | 6.4 |
| Robetta | 93.6 | 6.4 | 0 | 0 |
| trRosetta | 98.2 | 1.8 | 0 | 0 |
| Intfold | 84.4 | 11.0 | 1.8 | 2.8 |
| Phyre2 | 70.6 | 20.2 | 2.8 | 6.4 |
| RaptorX | 89.9 | 10.1 | 0 | 0 |

**
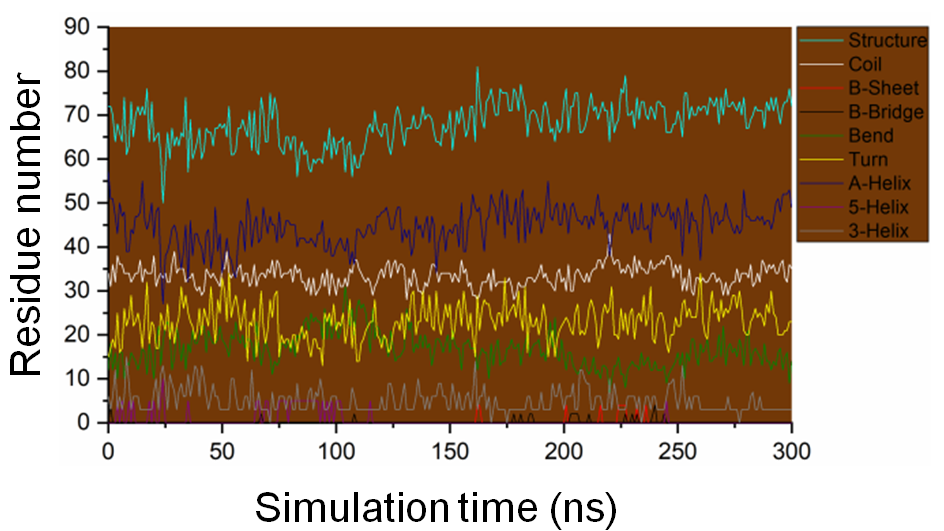
**

**Supplementary Figure S2.** The total number of residues involved with each secondary structure type in CbtA. Cyan, white, red, black, green, yellow, blue, purple, and gray curves represent total number residues involved in secondary structure, coil, β-sheet, β-bridge, bend, turn, α-helix, 5-helix, and 3-helix, respectively.

**
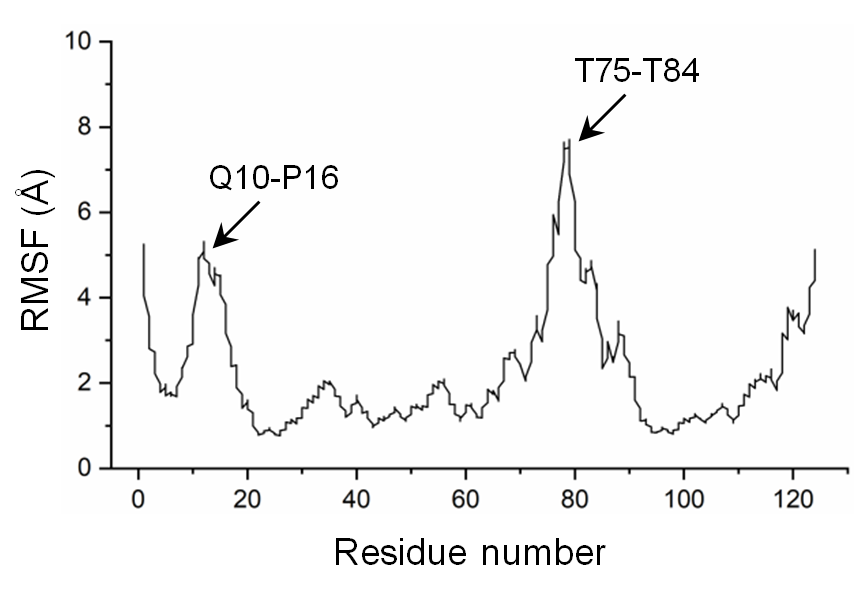
**

**Supplementary Figure S3.** Backbone RMSF of the CbtA model.

**
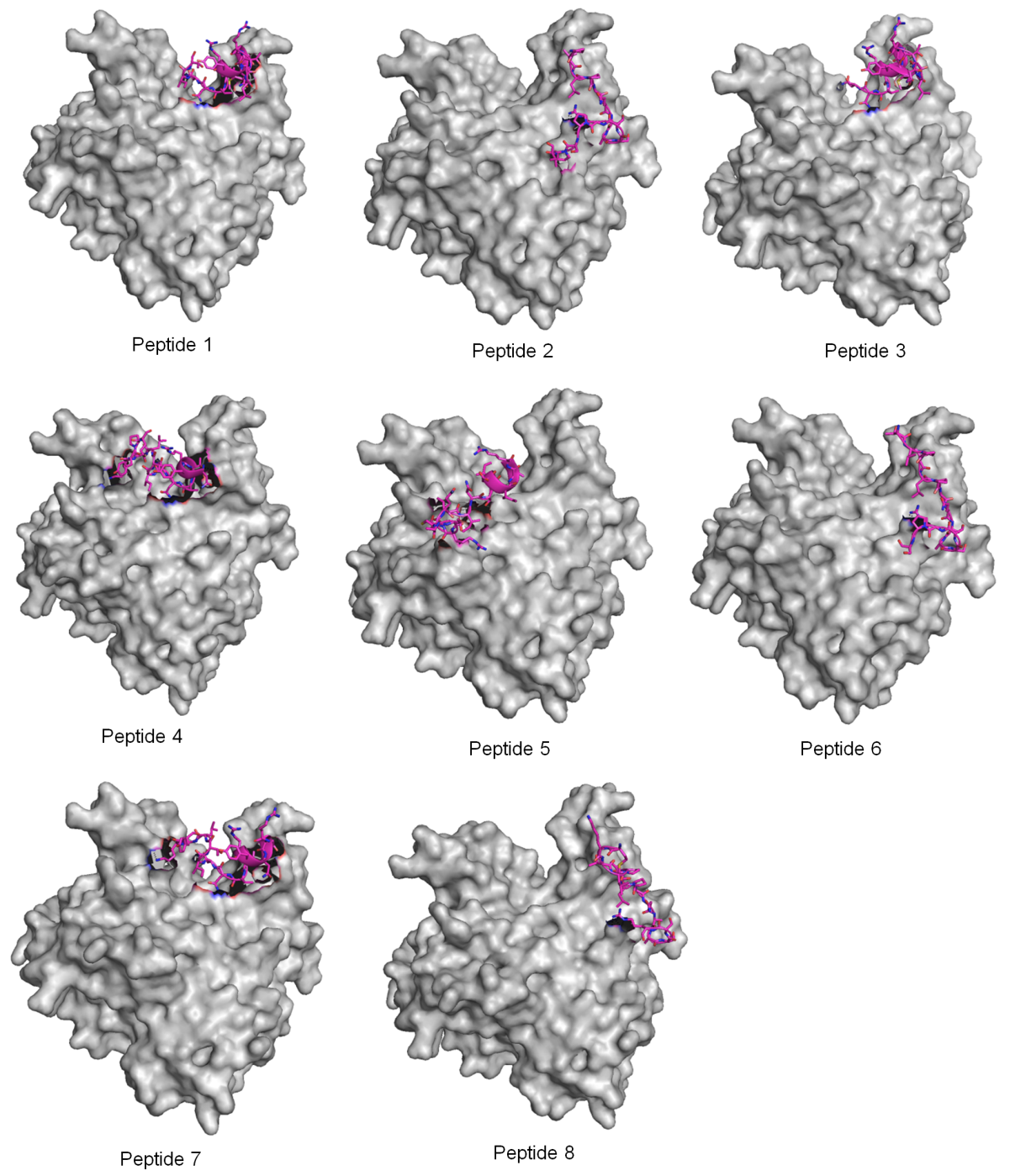
**

**Supplementary Figure S4.** Contacting sites of the eight predicted peptides on MreB. MreB is shown as gray surface. The peptides are colored in magenta.

.
